# Experimental demonstration of a quantum shutter closing two slits simultaneously

**DOI:** 10.1038/srep35161

**Published:** 2016-10-14

**Authors:** Ryo Okamoto, Shigeki Takeuchi

**Affiliations:** 1 Kyoto University, Department of Electronic Science and Engineering, Kyoto Daigaku-Katsura, Nishikyo-ku, Kyoto 615-8510, Japan

## Abstract

The interference between two paths of a single photon at a double slit is widely considered to be the most paradoxical result of quantum theory. Here is a new interesting question to the phenomenon: can a single shutter simultaneously close two slits by effectively being in a superposition of different locations? Aharonov and Vaidman have shown that it is indeed possible to construct a quantum shutter that can close two slits and reflect a probe photon perfectly when its initial and final states are appropriately selected. Here we report the experimental demonstration of their proposal overcoming the difficulty to realize a ‘quantum shutter’ by employing photonic quantum routers. The reflectance ratio of 0.61 ± 0.027 surpasses the classical limit with 4.1 standard deviation, shedding new light on the unusual physical properties of quantum operations. This experimental demonstration, where the strong measurement and non-local superposition seem co-existing, provides an alternative to weak measurements as a way to explore the nature of quantum physics.

The interference experiment using a single photon passing through a double slit^1^,^2^ proves the most paradoxical claim of quantum theory, “A particle can be in different places simultaneously^3^,^4^,^5^.” It is also well known that the interference fringe disappears when one monitors through which slit the photon actually passes^6^,^7^,^8^. This double-slit experiment continues to provide new insights into modern quantum physics; an example of this is the weak measurement to analyze the trajectory of a single photon^9^. Recently, Aharonov and Vaidman^10^ raised an interesting new question: can one quantum shutter being in a superposition of different locations close two or more slits and reflect the photons perfectly without disturbance? Surprisingly, they showed that the theoretical answer is yes, provided the quantum shutter is prepared in an appropriate preselected state and a particular final state of the shutter is postselected. However, as they predicted, an experimental demonstration has been prevented due to the difficulty of realizing a quantum shutter.

Here, we demonstrate the protocol proposed by Aharonov and Vaidman (AV03)^10^ using a photonic quantum circuit. As a quantum shutter, we used a shutter photon in a superposition state in modes that control two photonic quantum routers (PQRs) for the nonlinear interaction with a probe photon. The experimental results show that when the shutter photon is found in the appropriate final state, the input probe photon is reflected by the quantum shutter, with a probability exceeding the classical limit. By checking the coherence of the output probe photon, we also verified that the quantum superposition of the probe photon is not destroyed by the shutter. This experimental demonstration provides an alternative to weak measurements^11^ as a way to explore the unusual physical properties of preselection and postselection in quantum theory, and show counterintuitive aspect of quantum theory as have also been shown in three-box paradox^12^, Hardy’s paradox^13^, and quantum Cheshire-Cat^14^.

## Theory of the AV03 Protocol

**Figure 1:**
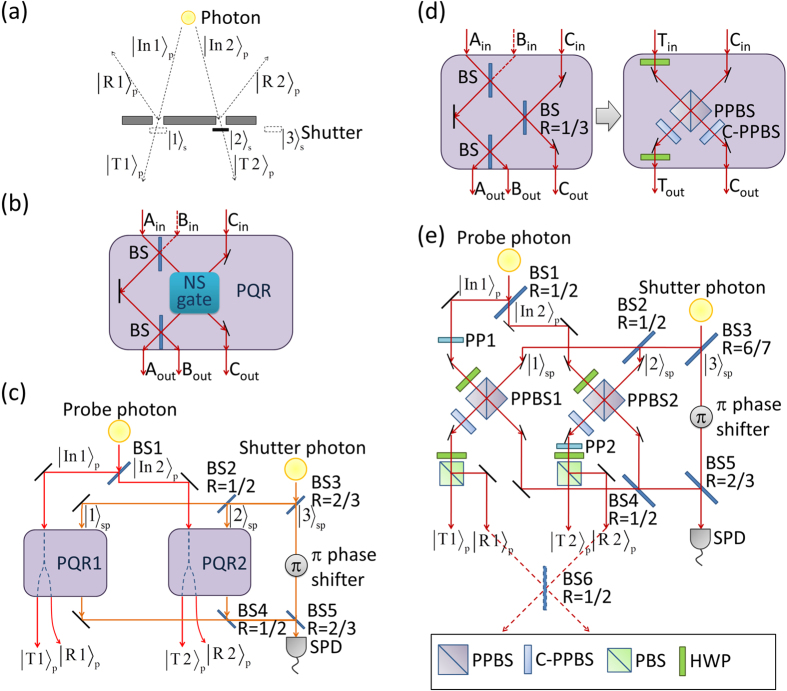
(**a**) Schematic for the AV03 protocol with two slits. 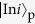 is the photonic state propagating in mode *i*. 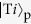 is the state of a probe photon where the photon passes through the slit *i*. 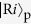 is the photonic state reflected by the shutter at slit *i*. 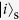 is the state of the shutter at the place *i*. (**b**) Photonic quantum router (PQR). A nonlinear sign shift (NS) gate is embedded in a Mach-Zehnder interferometer. BS: beam splitter. (**c**) Photonic scheme using the PQRs for implementing the AV03 protocol. 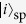 is the photonic state of the shutter photon propagating in mode *i*. SPD: single-photon detector. (**d**) Linear optical (LO) NS gate. The reflectance of the partially polarizing beam splitter (PPBS) for horizontally and vertically polarized photons is 1/3 and 1, respectively. The transmittance of the circular PPBS (C-PPBS) for horizontally and vertically polarized photons is 1 and 1/3, respectively^20^. (**e**) Photonic circuit for the scheme shown in (**c**) using linear optics NS gates. HWP: half-wave plate; PP: phase plate.

Figure 1(a) shows the protocol proposed by Aharonov and Vaidman (AV03) for the two-slit case. The state of the probe photon moving towards the two slits is


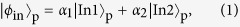


where 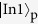 and 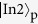 are the states of a photon moving toward slit 1 or slit 2, respectively. The quantum shutter is prepared (preselected) in the state


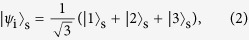


where 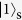 and 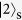 are states of a shutter located at slit 1 and slit 2, respectively, and 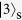 is the state of the shutter located at some specific place but different from slit 1 and slit 2. At slit 1 and slit 2, the photon is perfectly reflected when the shutter is at the same slit:


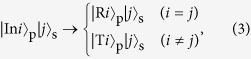


where *i* = 1, 2, *j* = 1, 2, 3, and 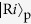 is the state of a probe photon reflected by the shutter at slit *i*. 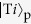 is the state of a probe photon where the photon passes through the slit *i*. Equation (3) satisfies the condition assumed by AV03^10^ that the photon bouncing on the shutter causes no measurable recoil. Then, after the interaction between the shutter and the photon (Eq. (3)), the joint quantum state of the photon and the shutter is


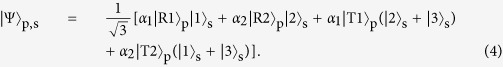


We will consider the following final state for the shutter:


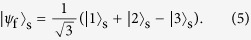


This final state is orthogonal to the state of the last two terms in Eq. (4). Therefore after the postselection, the photon state will have only reflected wave components 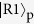 and 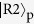 as follows:


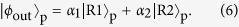


This means that the probe photon is completely reflected by the single quantum shutter, when the final state of the shutter is found in 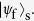 Note that the amplitudes and the coherence of the input state 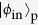 are maintained in the output state 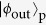. Note also that the probability of the postselection is 1/9.

## Experimental Realization of the AV03 Protocol

The crucial element for the demonstration of this protocol is the realization of a quantum shutter. For this purpose, we propose using a photonic quantum router (PQR), as shown in Fig. 1(b). The PQR consists of a two-mode nonlinear sign shift (NS) gate embedded in a Mach-Zehnder interferometer^15^,^16^, as shown in Fig. 1(b). When there is no control photon input to mode C_in_, the photon input to A_in_ is routed to mode A_out_. In contrast, the photon is routed to mode B_out_ when a control photon is input to mode C_in_. Note that when a superposition state is input to mode C_in_, the output state becomes entangled.

In order to implement the AV03 protocol, we propose a photonic scheme using the PQRs as shown in Fig. 1(c). In this scheme, the quantum shutter is represented by a shutter photon, and the interaction between the PQR and the probe photon (Eq. (3)). The superposition of the probe photon in Eq. (1) is prepared by BS1 in Fig. 1(c). The superposition state of the shutter, as given by Eq. (2), is prepared using BS2 and BS3 in Fig. 1(c). At the PQR, the interaction between the probe photon and the shutter photon occurs through its unitary transformation, as follows:


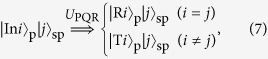


where *i* = 1, 2, and *j* = 1, 2, 3; this is exactly the same as the transformation given in Eq. (3). The projection of the joint quantum state of the probe photon and the shutter photon onto the final state 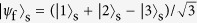 can be realized by detecting the shutter photon after the unitary transformation by BS4 and BS5, and use of the *π* phase shifter. Thus, when the shutter photon is detected by the single-photon detector (SPD), the probe photon is always found in the superposition state between 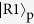 and 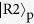 with the same probability amplitudes as the initial state (Eq. (1)), and it is never found in 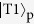 or 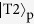. For the NS gate in the PQR (Fig. 1(b)), we may use a linear optical (LO) NS gate^17^,^18^,^19^,^20^,^21^,^22^ using two-photon interference at a beam splitter with reflectivity *R* = 1/3, as shown in Fig. 1(d). The operation of the LO-NS gate is successful when the number of photons in C_in_ is preserved in the output C_out_. In this case, the PQR can be implemented using a partially polarizing beam splitter (PPBS)^20^,^21^,^22^, where the modes A and B, respectively, correspond to the horizontal and vertical polarization of the target mode T. Thus, the quantum router changes the polarization of the probe photon if the shutter photon is present. Figure 1(e) shows the photonic circuit for the scheme (Fig. 1(c)) using the LO-NS gates. BS6 is used to check if the coherence of the reflected probe photons is preserved. The reflectance of BS3 (6/7) is different from that (2/3) shown in Fig. 1(c) because it is necessary to compensate the probability amplitudes after the LO-NS gates.

As explained above, the LO-NS gate has operated successfully when the number of photons in the control mode C_in_ is preserved in the output mode C_out_ (Fig. 1(d)). However, in the photonic circuit shown in Fig. 1(e), we are not able to monitor the exact number of photons at each C_out_ of the two gates, but we are able to monitor the total number of photons output from the C_out_ of the two LO-NS gates. Thus, there are unwanted cases where the total number of photons output from the two C_out_s is the same as the total number of photons input to the two C_in_s, but the numbers of photons input to C_in_ and output from C_out_ are not preserved at each LO-NS gate.

The effect of the unwanted events appears as residual terms, as seen in Eq. (15) in Methods section. However, the terms are sensitive to the phase *δ* due to the optical path-length difference and this is very difficult to analyze. We have found that we can average this effect and make it insensitive to *δ* by uniformly randomizing the phase, as shown in Eq. (16) in Methods section. For this purpose, we add the same random phase using the phase plates (PP1 and PP2) to both of the input modes (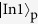, 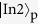) of the probe photon, while preserving the relative phase of the input probe photon (Fig. 1(e)). However, due to the residual components, there are cases where the probe photons are found in the transmitted modes (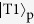 or 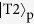) even when the shutter photon is detected by the single-photon detector.

In order to characterize the performance of our scheme, we adopt the reflection ratio *P*_*R*_ = *N*_*R*_/(*N*_*T*_ + *N*_*R*_), where *N*_*R*_ is the number of reflected probe photons, and *N*_*T*_ is the number of transmitted probe photons when the shutter is found in 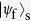. Let us consider the case where the number of the slits is *N*. When the probe photon is uniformly distributed among the slits, an ideal quantum shutter can perfectly reflect the probe photon when the shutter is found in the appropriate final state. Hence, *P*_*R*_ = 1. On the other hand, a classical shutter, which can be set to any one slit, reflects the photon with a probability 1/*N*. Thus, when the probe photon is uniformly distributed among the slits and the photon bouncing on the shutter causes no measurable recoil, the reflection ratio for the classical case is *P*_*R*_ = 1/*N*. This is the largest possible value of the reflection ratio for the classical case (see Methods section). Thus, the shutter is nonclassical if the reflection ratio is larger than 1/*N*. For *N* = 2, the condition that exceeds this classical limit is *P*_*R*_ > 1/2. As is described in detail in Methods section, we found that the scheme shown in Fig. 1(e) can exceed the classical limit, up to *P*_*R*_ = 2/3.

**Figure 2:**
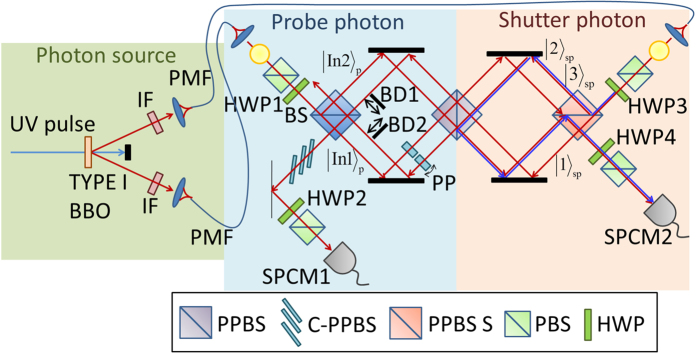
Schematic of the experimental setup. The shutter photon mode 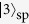 is realized using the vertical polarization mode 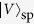 (blue line). The reflectance of PPBS S for horizontally and vertically polarized photons is 1/2 and 1, respectively. PMF: polarization maintaining fiber; SPCM: single-photon counting module; IF: interference filter; BBO: *β*-Barium borate; BD: beam dumper.

For an experimental realization of the photonic circuit shown in Fig. 1(e), there is a critical obstacle to overcome. As is explained in Methods section in detail, the photonic circuit consists of two multipath interferometers: one is for the probe photon, and the other is for the shutter photon. Furthermore, these multipath interferometers are connected by PPBS1 and PPBS2. The difference between the path lengths of the multipath interferometers must be accurate to within a few nanometers; this is quite challenging. We overcame this problem by using a displaced Sagnac architecture^23^,^24^, as shown in Fig. 2. The experimental setup and how the photonic circuit shown in Fig. 1(e) can be converted to the setup are explained in Methods section in detail. During the accumulation time for each measurement, for the randomization of the phase *δ*, the two phase plates provided over one hundred randomly selected different phases.

## Results and Discussion

First, we verified that the reflection ratio *P*_*R*_ surpasses the classical limit 1/2. We counted the number of coincidences between the two single-photon counting modules (SPCMs) shown in Fig. 2 with changing the angle of HWP2 for horizontal (transmitted) and vertical (reflected) polarization output modes. The observed coincidence counts for the transmitted photons and the reflected photons were *N*_*T*_ = 313 ± 18 and *N*_*R*_ = 492 ± 22, respectively, during a two-second period. Thus, the ratio *P*_*R*_ is 0.61 ± 0.027, which surpasses the classical limit of 1/2 with a standard deviation of 4.1. This provides clear evidence of the nonclassical effect of the AV03 protocol.

**Figure 3:**
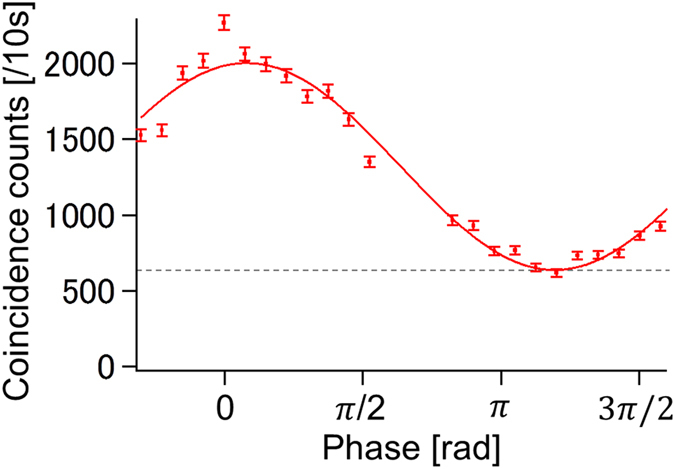
Interference fringe for the reflected probe photons. The coincidences of the probe and shutter photons are plotted as a function of the phase in the probe photon interferometer. The phase is calculated from a single-photon experiment. The dashed line corresponds to the calculated counts given by the residual state. The error bars are determined by assuming that the count follows the Poisson distribution.

Next, we checked if the coherence of the probe photon was preserved. The phase between the modes (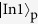 and 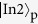) of the probe photon was changed by rotating the angle of a glass phase plate (PP in Fig. 2). Figure 3 shows the interference of the reflected modes (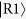 and 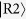) of the output probe photon after passing through BS6 in Fig. 1(e), while the phase is changed between the two modes of the photon state of the input probe (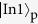 and 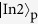). The visibility of the observed fringe is 52 ± 2%, and it is limited by the residual components (dashed line). If we compensate for the effect of the residual components, the visibility becomes 103 ± 6% and shows that the reflected probe photon almost perfectly maintains the coherence of the input probe photon, as predicted by the original AV03 protocol. Note that the interference confirms that the two shutter positions are in superposition, since a local reflection would make interferences impossible. Thus Fig. 3 provides independent evidence for the quantum nature of the effect.

**Figure 4:**
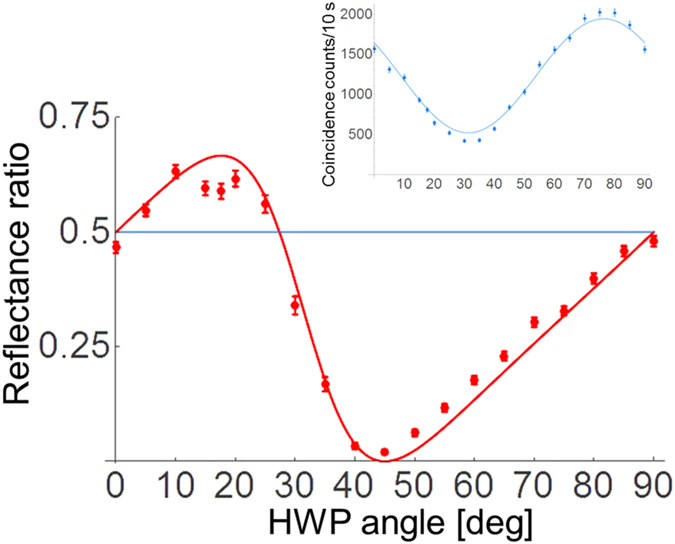
Reflection ratio *P*_*R*_ in terms of the HWP angle, which changes at the shutter photon output (Fig. 2). The error bars are determined by assuming that the count follows the Poisson distribution. The solid curve corresponds to the reflection ratio, which is derived theoretically. The classical limit 1/2 of the reflection ratio is indicated by a solid line. Inset: blue dots show the observed total counting rate *N*_*T*_ + *N*_*R*_ in terms of the HWP angle. The solid curve corresponds to the theoretical curve using Eq. (17) in Methods section, where *N* was treated as a fitting parameter.

Finally, we validated that our implementation can faithfully realize the operations used in the AV03 protocol. By rotating HWP4 at the shutter photon output in Fig. 2, we are able to tune the relative amplitude and phase between the state 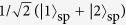 and 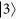 as 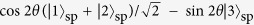, where *θ* is the angle of the HWP. Figure 4 shows the reflection ratio *P*_*R*_ as the postselected state is varied. The solid curve corresponds to the theoretical calculation. The maximum value of the reflection ratio *P*_*R*_ is at *θ* = 17.63 deg, when the shutter is postselected in the state proposed by AV03 (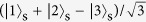). The reflection ratio *P*_*R*_ becomes 0 at the HWP angle of 45 deg, confirming that almost perfect transmission is achieved when there is no shutter photon in mode 1 or 2. When *θ* = 0 or 90 deg, the postselected state of the shutter is 
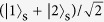
. Thus, the shutter is superposed on just two slits, and the theoretical value of *P*_*R*_ = 1/2. Note that this case should also preserve the coherence between mode 1 and 2. The reflection ratio of 1/2 confirms that the shutter state 3 is essential for the non-classical shutter interference. The experimental results are in good agreement with the theoretical curve for all of the postselected states. The deviation at the HWP angle about 15 degrees to 25 degrees may have been caused by the small leakage of photons from the closed shutter. Note that the experimentally obtained total counting rate *N*_*T*_ + *N*_*R*_ (blue dots, Fig. 4 inset) agreed well with the theoretical prediction (blue curve, Fig. 4 inset).

Here let us discuss the success rate of the photonic circuit (Fig. 1(e)) we proposed. We define the success rate to be the rate at which the probe photon in the reflected state and the shutter photon in the final state are simultaneously detected. In the original AV03 protocol^10^, the success rate for the case with *N* slits is 1/(2*N* − 1)^2^. Thus, when *N* = 2, the success rate is 1/9. On the other hand, the success rate of our experimental scheme is 2/63. This is due to the two reasons. The first is that we used the photonic router with non-unity success probability. Due to the nature of the quantum phase gate we adopted^17^, the probability amplitude of 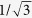 is multiplied to both the signal photon and the shutter photon when the photons path through the photonic routers. For this reason, the probability amplitude of 1/3 is multiplied to each components of the joint quantum state in Eq. (4) and thus the success probability of the shutter operation is 1/9. Thus, the success rate will become 1/81 (“the success probability for shutter operation = 1/9” times “the postselection probability of AV03 = 1/9”). In our implementation, we improved this rate from 1/81 to 1/63 by optimizing the probability amplitudes of the shutter photon. The second reason is that there are “unwanted events” due to the existence of the residual terms. The probability of having such events is 1/63. Thus, the total success rate is 2/63.

In our demonstration, the reflection ratio *P*_*R*_ is limited to 2/3 due to the residual terms, which emerge because we cannot distinguish some unsuccessful operations of the LO-NS gates. Alternatively, we could eliminate the residual terms and make the reflectance ratio *P*_*R*_ equal unity by using the heralded type of NS gate, which has been demonstrated previously^25^,^26^.

## Conclusion

In conclusion, we have proposed the photonic scheme to implement AV03 protocol and designed the photonic quantum circuit using the LO-NS gates, which has been successfully demonstrated. The experimental results show that when the shutter photon is found in the appropriate final state, the input probe photon is reflected by the quantum shutter, with a reflectance ratio of 0.61 ± 0.027 exceeding the classical limit of 0.5 with 4.1 standard deviation. We also verified that the reflected probe photon almost perfectly maintains the coherence of the input probe photon, as predicted by the original AV03 protocol. This counterintuitive result provides further evidence that quantum effects cannot be traced back to well-defined elementary events, but require a more fundamental regard for the relations between seemingly separate possibilities as well as other counterintuitive phenomena^12^,^13^,^14^. We would like to stress that our scheme can be applied to any quantum system other than photonic qubits, e.g. superconducting qubits, in which the function of the PQR can be realized.

The AV03 protocol demonstrated here has revealed that a combination of preselection and postselection of the quantum states can be used to realize tasks that are completely counterintuitive. We believe this demonstration sheds new light on the theory and applications of preselected and postselected quantum states. One interesting feature of this experimental demonstration is that the flow of a signal photon in multiple (*N*) paths (multiple locations) is controlled using just ONE control photon (shutter photon), by changing the postselected state as shown in Fig. 4, with the cost of the postselection probability. We think our experiment may provide a new way to control the flow of multiple photons in the multi-path photonic network, which is attracting lots of attention for boson-sampling^27^,^28^,^29^,^30^, quantum random walks^31^,^32^,^33^, and quantum simulation^34^.

## Methods

### Classical limit of the reflectance ratio

Here, we explain in detail the classical limit of the reflectance ratio. If the shutter is a classical object, it cannot be in a state of superposition. Thus, in general, the classical shutter state becomes a mixed state, as follows:


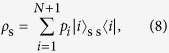


where *N* is the number of slits, *p*_*i*_ is the probability that the shutter is located at slit *i* and 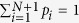. On the other hand, the state of the probe photon is given by


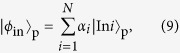


where 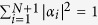. The joint quantum state is


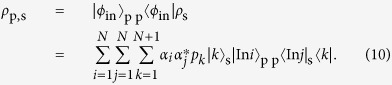


After the interaction between the shutter and the probe photon, the above joint state transforms to


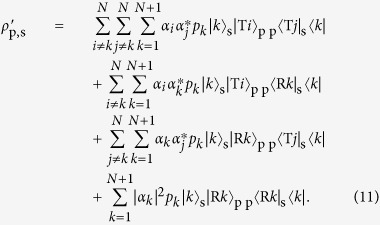


The probability that the probe photon will be reflected by the shutter is


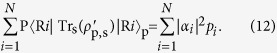


When the probe photon is incident into *N* slits with uniform probability amplitudes, |*α*_*i*_|^2^ = 1/*N*, and thus,


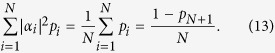


Therefore, the classical limit of the reflection ratio becomes *P*_*R*_ = 1/*N*.

### Linear optical NS gate and the residual state

**Figure 5:**
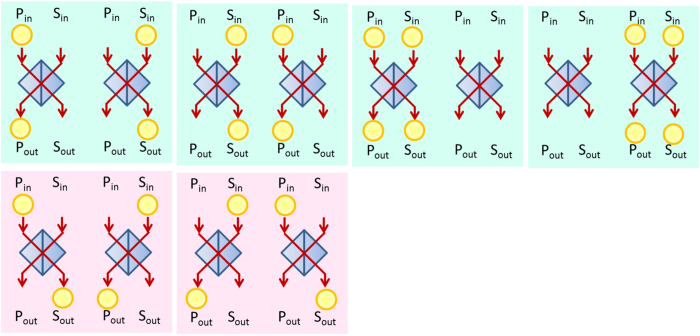
Input-output combinations of the probe and shutter photons for two LO-NSs.

For the NS gate in the PQR, we used a linear optical (LO) NS gate with two-photon interference at a beam splitter with reflectivity *R* = 1/3, as shown in Fig. 1(d). The operation of the LO-NS gate is successful when the number of photons input to C_in_ is preserved in the output C_out_. As shown in Fig. 1(d), we used two LO-NS gates in our experimental scheme. Figure 5 shows the possible combinations of input and output for the probe and shutter photons. Since the probe photon and the shutter photon are injected from P_in_ and S_in_ respectively, there are four combinations for the input photons. The both LO-NS gates are successful when the number of photons in each S_in_ is preserved in each S_out_ (blue panels in Fig. 5). However, there are two unsuccessful combinations (red panels in Fig. 5), because the sum of the photon numbers in the output shutter (control) modes can only be measured by the single-photon detector. As a result, the remaining two combinations add two residual terms to Eq. (4), as follows:


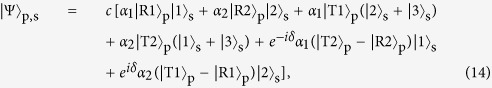


where *c* is the normalization coefficient and *δ* is the phase between the two successful events (left two blue panels in Fig. 5) and the two unsuccessful events (red panels in Fig. 5). For convenience, we rewrite Eq. (14) as


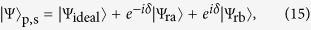


where 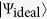 has the same terms as the ideal state shown in Eq. (4) except for the normalization, and the other two states correspond to the residual terms: 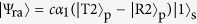 and 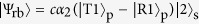. However, the residual terms are sensitive to the phase *δ* due to the optical path-length difference in Eq. (15), which is very difficult to analyze as it is. We have found that we can average this effect and make it insensitive to *δ* by uniformly randomizing the phase.

After this operation, the pure state in Eq. (15) transforms to a mixed state


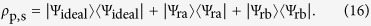


The state after the postselction of the shutter is given as follows:


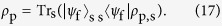


Thus, the probability *p*_T_ that we find the transmitted probe photon is


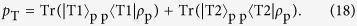


By substituting Eq. (16) for Eq. (17), we obtain


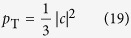


Similarly the probability *p*_R_ for the reflected probe photon is


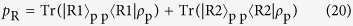



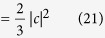


By these probabilities we obtain the reflectance ratio


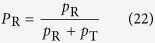



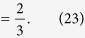


Note that *P*_R_ is independent from *α*_1_ and *α*_2_.

### Implementation of the proposed photonic circuit using an inherently stable displaced Sagnac architecture

Here, we explain our implementation of the experimental setup shown in Fig. 2 for the photonic circuit shown in Fig. 1(e). The photonic circuit can be considered to be two multipath interferometers coupled by PPBS1 and PPBS2. One of the multipath interferometers is for the probe photon, which is split by BS1 and become a superposition state between 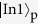 and 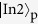, and, after the interactions at PPBS1 and PPBS2, the mode components of the probe photon in 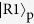 and 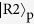 are interfered at BS6. The other multipath interferometer is for the shutter photon, which become a superposition state in modes 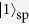, 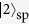, and 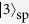 by BS2 and BS3, and after the interaction with the probe photon at PPBS1 and PPBS2, they are merged by BS4 and BS5.

**Figure 6:**
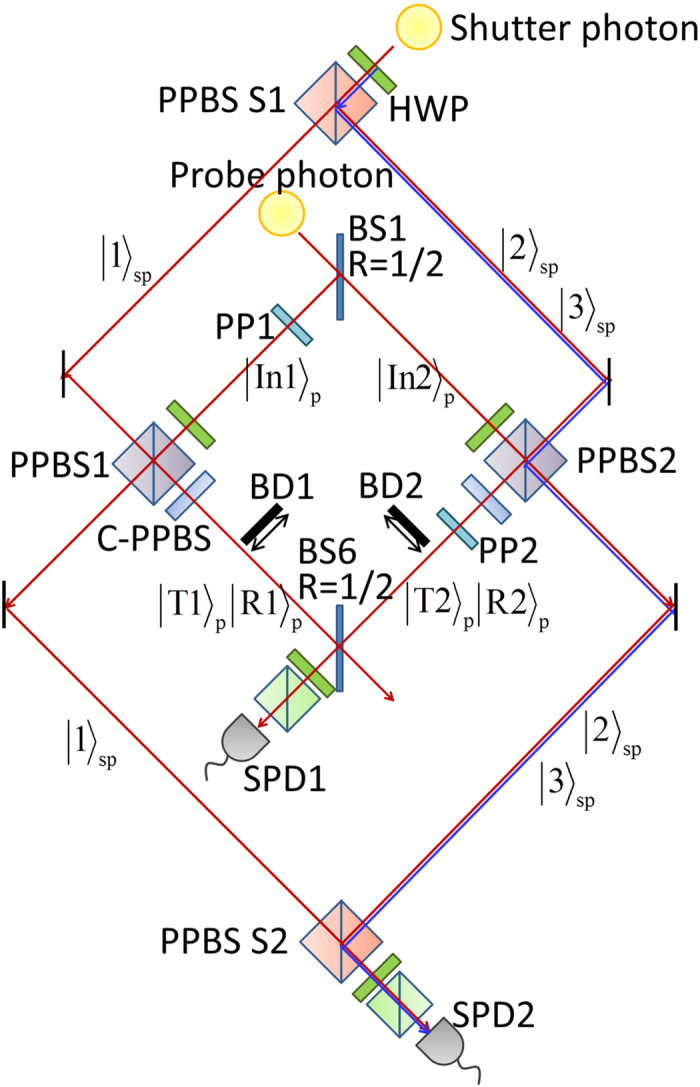
Photonic circuit using the PPBS Ss. The shutter photon mode 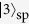 is realized using the vertical polarization mode 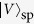 (blue line). The reflectance of PPBS S for horizontally and vertically polarized photons is 1/2 and 1, respectively. SPD: single-photon detector; BD: beam dumper.

This situation can be seen more clearly in Fig. 6, which is an implementation of the optical circuit shown in Fig. 1(e). In this implementation, the modes 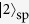 and 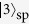 (blue line) of the shutter photon are realized by the two orthogonal polarization modes of one optical path. Thus, the three-path interferometer for the shutter photon shown in Fig. 1(e) is now implemented as a two-path Mach-Zehnder like interferometer that uses PPBS S1 and PPBS S2. Note that we can check the flux of the probe photons in 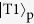 (or 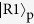) when we block the 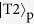 components by inserting a beam dumper, shown as BD2 in Fig. 6.

Now we have a problem to be solved. We need to stabilize the optical path differences of these coupled interferometers to an accuracy of within a few nanometers for the duration of this series of experiments (hours). For this purpose, we adopted a displaced Sagnac architecture^23^,^24^ for the interferometers (Fig. 2). The two optical paths in a displaced Sagnac interferometer share the same optical component, and thus small drifts or fluctuations in the optical component are automatically canceled. Since the optical paths are folded in the Sagnac interferometers, the PPBS in Fig. 2 fills the same function as the combination of PPBS1 and PPBS2 in Fig. 6. Similarly, PPBS S in Fig. 2 works as PPBS S1 and PPBS S2 in Fig. 6, and BS in Fig. 2 works as BS1 and BS6 in Fig. 6. In this way, we were able to realize the photonic circuit shown in Fig. 1(e) for the AV03 protocol as a compact and stable form. When the shutter photon is found in the state 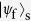, the number of reflected (transmitted) probe photons in mode 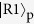 (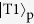) can be measured by inserting the beam dumper (BD2) into the interferometer and counting the coincidence detection events between SPCM1 and SPCM2. Similarly, the number of probe photons in 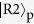 or 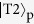 can be measured by inserting BD1. When neither BD1 nor BD2 is inserted, we can measure the result of the interference between the output probe photon states in 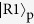 and 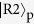. In this case, we change the phase difference between 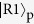 and 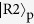 by rotating the phase plate (PP) in the interferometer. The offset between the phases of the polarization modes 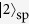 and 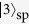 are adjusted by a birefringent plate (not shown).

### Photon source

For the probe and shutter photons, we used pairs of photons generated via type-I spontaneous parametric down-conversion as shown in Fig. 2. The pump laser pulses (76 MHz at 390 nm, 200 mW) pass through a beta-barium borate crystal (1.5 mm). The pairs of photons were delivered to the optical quantum circuit through the polarization-maintaining fibers (PMFs).

### The dependence of *N*_*T*_ + *N*_*R*_ on the half-wave-plate angle *θ*

As shown in the inset of Fig. 4, the total counting rate *N*_*T*_ + *N*_*R*_ depends on the postselected state of the shutter. Here, we discuss this dependence of the total counting rate on the half-wave-plate (HWP) angle *θ*. In our experiment, we changed the postselected state by varying the angle *θ* of HWP4 at the shutter photon output shown in Fig. 2. The state 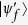 used for the postselection is 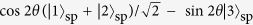. By substituting the above postselected state 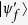 into 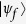 in Eq. (17), the probability of finding the probe photon either transmitted or reflected by the shutter is given as follows:


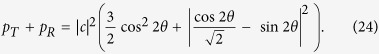


The total conditional counting rate is thus given by


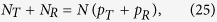


where *N* is a constant value corresponding to the input probe photon number. *N*_*T*_ + *N*_*R*_ has minimum and maximum values at 31.3 and 76.3 degrees, respectively.

## Additional Information

**How to cite this article**: Okamoto, R. and Takeuchi, S. Experimental demonstration of a quantum shutter closing two slits simultaneously. *Sci. Rep*. **6**, 35161; doi: 10.1038/srep35161 (2016).
